# Age-Related Deterioration of Mitochondrial Function in the Intestine

**DOI:** 10.1155/2020/4898217

**Published:** 2020-08-18

**Authors:** Anna M. Schneider, Mihriban Özsoy, Franz A. Zimmermann, René G. Feichtinger, Johannes A. Mayr, Barbara Kofler, Wolfgang Sperl, Daniel Weghuber, Katharina Mörwald

**Affiliations:** ^1^Department of Pediatrics, Paracelsus Medical University, Salzburg, Austria; ^2^Research Program for Receptor Biochemistry and Tumor Metabolism, Department of Pediatrics, Paracelsus Medical University, Salzburg, Austria

## Abstract

Aging is an important and inevitable biological process in human life, associated with the onset of chronic disease and death. The mechanisms behind aging remain unclear. However, changes in mitochondrial function and structure, including reduced activity of the mitochondrial respiratory chain and increased production of reactive oxygen species—thus oxidative damage—are believed to play a major role. Mitochondria are the main source of cellular energy, producing adenosine triphosphate (ATP) via oxidative phosphorylation. Accumulation of damaged cellular components reduces a body's capacity to preserve tissue homeostasis and affects biological aging and all age-related chronic conditions. This includes the onset and progression of classic degenerative diseases such as cardiovascular disease, kidney failure, neurodegenerative diseases, and cancer. Clinical manifestations of intestinal disorders, such as mucosal barrier dysfunction, intestinal dysmotility, and chronic obstipation, are highly prevalent in the elderly population and have been shown to be associated with an age-dependent decline of mitochondrial function. This review summarizes our current understanding of the role of mitochondrial dysfunction in intestinal aging.

## 1. Introduction

Mitochondria are double-membrane organelles that contain their own circular double-stranded DNA (mtDNA) [1, 2]. They convert carbohydrates and fats, which are the main sources of cellular energy, into adenosine triphosphate (ATP) by oxidative phosphorylation (OXPHOS) via the mitochondrial respiratory chain (RC). Besides ATP production, mitochondria contribute to heme and iron-sulfur cluster biogenesis, calcium homeostasis, *β*-oxidation of fatty acids, cellular differentiation, cell death regulation, and control of the cell cycle [3, 4].

Recent data support the notion that declining mitochondrial function is a crucial element of human aging and its associated frailty syndrome [5–13]. Accumulation of damaged cellular components causes a decline in physiological function and a reduced capacity to maintain tissue homeostasis, which plays a pivotal role in the process of aging [1, 14–17]. Changes in mitochondrial function and structure, including a reduction in mitochondrial OXPHOS activity, increased production of reactive oxygen species (ROS), and damage to DNA, proteins, and lipids, characterize aging [18]. Additionally, accumulation of mtDNA mutations is a genetic correlate of human aging [19]. Previous studies in animal models showed that mtDNA mutations lead to premature aging and reduced life span [7, 20–22].

Clinical manifestations of intestinal disorders, such as mucosal barrier dysfunction, intestinal dysmotility, and chronic obstipation, are highly prevalent in the elderly population. Previous reports have suggested that mtDNA mutations increase exponentially with age and lead to a reduction or complete loss of expression of OXPHOS complexes in intestinal tissue [1, 5, 6, 23]. Additionally, alterations in mitochondrial morphology, intestinal structure, permeability, and motility have been described with increasing age, which are linked to clinical manifestations such as chronic constipation and mitochondrial neurogastrointestinal encephalopathy- (MNGIE-) like signs and symptoms [24–36]. This review summarizes current understanding of the role of mitochondrial dysfunction in intestinal aging including structural changes, mtDNA mutations, decline in respiratory chain function, increased ROS production, and telomere dysfunction.

## 2. Materials and Methods

This review was conducted in line with the Preferred Reporting Items for Systematic Reviews and Meta-Analysis (PRISMA) guideline [37].

### 2.1. Data Source/Search Strategy

Studies focusing on the role of mitochondria in the aging intestine were retrieved from PubMed. Searches were conducted from August 2018 to March 2020. The following predefined search terms were used: mitochondria, respiratory chain, OXPHOS, complexes, electron transport complexes, mitochondrial structure, oxidative stress, free radicals, ROS, energy metabolism, mtDNA, mutation, aging, frailty, biological vulnerability, telomere, stem cells, epithelial cells, homeostasis, mutation, immunohistochemistry, adolescent, adults, human aging, gastrointestinal tract, intestine, colonic mucosa, colon, mitochondrial dysfunction, gastrointestinal motility, intestinal permeability, constipation, intestinal barrier dysfunction, mitochondrial neurogastrointestinal encephalopathy (MNGIE), and antioxidants.

### 2.2. Study Selection

All papers were screened with regard to their titles and abstracts. Furthermore, the references listed in the studies cited in the present report were scrutinized to obtain further reports suitable for inclusion.

Articles not written in English were excluded. All types of study designs were included.

**Figure 1:**
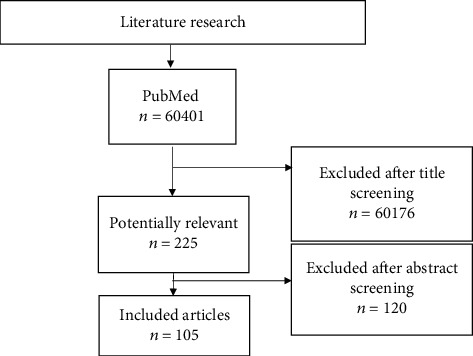
Flow chart presenting the screening and selection process.

Titles and abstracts of potentially relevant articles were screened, and full-text articles were screened by two independent examiners for inclusion in the review. All selected references were then extracted to EndNote software version X9 (Thompson Reuters; New York, USA). The selection process is pictured in Figure 1.

## 3. Mitochondria

Mitochondria are composed of four compartments: (a) the mitochondrial matrix containing the mitochondrial genome, its transcription and translation machinery, and enzymes involved in *β*-oxidation and the citric acid-cycle; (b) the inner membrane and its embedded OXPHOS complexes which generate ATP for cellular processes; (c) the outer membrane containing proteins involved in cell death and protein import as well as voltage-dependent anion channels (VDAC); and (d) the intermembrane space housing intermediate products of OXPHOS [3, 38]. The degradation of dysfunctional mitochondria that lack membrane potential is accomplished by a process known as mitophagy via the autophagic pathway, which is regulated by mitochondrial fission and fusion [10, 39, 40]. This process ensures mitochondrial quality control. A disruption may result in impairment of other cellular organelles [40, 41]. Each cell contains multiple mitochondrial genomes. Thus, mutations may lead to a mixture of mutant and wild-type copies of mtDNA in the same cell, which is known as heteroplasmy and is a hallmark of mitochondrial disease. The state of all mtDNA copies being the same is known as homoplasmy [3, 5, 23, 38].

Energy, which is needed for many cellular processes, is provided by mitochondria via OXPHOS catalyzed by the complexes of the RC and ATP synthase [42, 43].

## 4. Role of Mitochondria in the Aging Process

**Figure 2:**
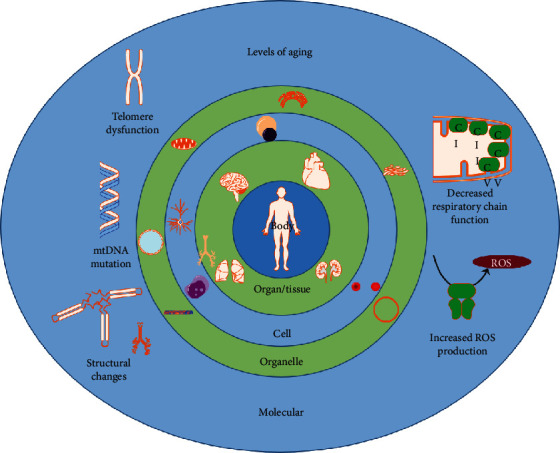
Common theories and definitions of aging. Aging cannot be explained by a single theory. There are multiple mechanisms on many levels, including not only the cellular and molecular level but also tissues and organ systems, contributing to the process of aging [46, 47]. Several theories exist: On the body/organism's level, aging is defined as an increase in mortality as it progresses throughout its lifecycle and increases its chronological age [48]. On the molecular level, increased ROS production, decreased respiratory chain function, structural mitochondrial changes, mtDNA mutation, and telomere dysfunction are important points associated with aging and the main discussed points in this review (Section 5). Harman first hypothesized the free radical theory of aging in 1956 [49]. He postulated that organisms age because cells accumulate free radical damage over time, and thus free radicals, due to the macromolecular damage they are exposed to, may be a potential cause of aging [49]. The cross-linking theory, also referred to as the glycosylation theory of aging, was proposed by Johan Bjorksten in 1942 and states that an accumulation of cross-linked proteins damages cells and tissues, slowing down bodily processes resulting in aging. A decline in mitochondrial quality and activity has been associated with normal aging and correlated with the development of a wide range of age-related diseases—the mitochondrial decline theory [50–52]. The membrane theory of aging was first described in 1994. According to this theory, it is the age-related changes of the cell's ability to transfer chemicals, heat, and electrical processes that impair it [53].All theories over all different levels influence each other. In example, mitochondria in the organelle level are the main site of ROS generation and when mortality on the organism level is higher, the more ROS production on the molecular level happens.

The number of elderly individuals has been rising in the last several decades, especially in Western countries [44]. In contrast, the proportion of young individuals is shrinking. This ongoing shift in population age structure means that more and more of the human population will be burdened with aging-related illnesses. Aging is characterized by a progressive decline in physiologic function due to a wide range of modifications impacting the structure and function of tissues in the body. Recent studies support the concept that alterations in mitochondria and mtDNA, which include structural and functional changes, play an important role in this degenerative process [45] (Figure 2).

### 4.1. Structural Changes in Mitochondria

Structural alterations of mitochondria have been described in different tissues of various species across a spectrum of ages. Examples include mouse tissue from the heart, skeletal muscle, and optic nerve and human tissue from the retina and skin [54–58]. Electron microscopic analysis of cardiomyocytes in aged rats showed disorganized and degenerated mitochondria with fewer and shorter cristae, as well as irregular assembly [54, 55]. Mitochondria in the optic nerve axons of aged mice displayed various abnormalities, including expanded mitochondrial volume but reduced numbers of the organelle [56]. Furthermore, skeletal muscle in aged mice exhibited larger and less circular subsarcolemmal mitochondria and longer and more branched intermyofibrillar mitochondria compared to those of younger animals [57]. In the nematode *C. elegans*, age-dependent morphological changes in body wall muscles were observed, revealed as fragmentation of the tubular mitochondrial network with increasing age, accompanied by a reduction in mitochondrial volume [59]. As age-associated mitochondrial dysfunction may contribute to vascular disease, the structure of mitochondria in freshly isolated myocytes from rat cerebral resistance arteries was analyzed, showing age-related changes in mitochondrial size [60]. A transmission electron microscopy study comparing the synapses and synaptic mitochondria of the CA1 region in young-adult (10 months of age) and old (22 months of age) male Fischer rats showed significant age-related synaptic loss, associated with mitochondrial structural damage in both pre- and postsynaptic compartments [61]. Most of this morphological damage includes type II damage (swollen, homogenized, and whirled cristae) and type III damage (crista homogenization and fragmentation in a significantly swollen electron-lucent matrix) [61].

With regard to human tissue, aged human retinal pigment epithelium showed significant reductions in the number and area of mitochondria, as well as loss of cristae and matrix density [58]. A study on human skin *in vivo* showed a significantly more fragmented network with smaller mitochondrial clusters in keratinocytes in old skin compared to keratinocytes in young skin [62].

### 4.2. Accumulation of mtDNA Mutations

It has been suggested that accumulation of mtDNA mutations in different tissues plays a major role in the process of aging [7, 21, 22, 63–66]. mtDNA mutator mice expressing a defective mitochondrial DNA-polymerase *γ* showed an increase in point mutations and deletions, leading to significant premature aging and reduced life span [7]. An increased load (3-5 times) of somatic mtDNA mutations in different tissues, like brain, heart, and liver, equally affecting all three codon positions, was also observed in mtDNA mutator mice [7]. Until the age of 25 weeks, these mice had a normal phenotype, but thereafter, a variety of aging phenotypes was observed prior to physiological aging of wild-type mice. Kyphosis, alopecia, weight loss, sarcopenia, presbycusis, osteoporosis, reduced subcutaneous fat, anemia, spleen enlargement, increased heart weight, left ventricle hypertrophy, and reduced fertility were seen earlier in mtDNA mutant mice as compared to wild-type mice [7, 20–22]. Sectioning of heart muscle tissue and immunohistochemical staining showed cytochrome-c oxidase (COX; complex IV of OXPHOS) deficiency in some cardiomyocytes, comparable to aging human hearts [7, 67]. Moreover, measurement of OXPHOS enzyme activities in the hearts of mtDNA mutator mice expressing defective mitochondrial DNA-polymerase *γ* demonstrated a decline in OXPHOS function and mitochondrial ATP production [7]. In addition, mtDNA mutations were also detected in the brain and duodenum of these mice; about 20% of cells in the duodenum were COX-negative, and the number of point mutations was increased about 100-fold compared to wild-type mice [22].

In addition to complex IV, OXPHOS complexes I and III were also reduced in heart and liver mitochondria of mtDNA mutator mice. This deficiency of multiple complexes can be explained by point mutations in mtDNA resulting in unstable OXPHOS complexes, as the nucleus-encoded subunits were also degraded [21].

### 4.3. Decline in Mitochondrial Respiratory Chain Function

Several studies suggest that mitochondrial OXPHOS activity in different human organs (e.g., extraocular muscle, skeletal muscle, liver, heart, and brain) deteriorates with age [8, 67–78]. Specifically, a reduction in the activity of the OXPHOS complexes I and IV was found to be associated with aging [79]. Immunohistochemical staining showed COX-negative muscle fibers in extraocular muscles, limb muscles, and the diaphragm and that COX deficiency increases with increasing age [8, 69]. Moreover, a decline in OXPHOS function has been reported in human brain tissue and the liver [70, 72]. COX-deficient muscle fibers were first observed in the third decade of life and increased with age [69]. In addition to complex IV, a decrease in complex I activity in human skeletal muscle was seen [71]. With regard to the brain, a significant age-related decrease in COX activity was observed in four regions of the brain [70]. Furthermore, mitochondrial activity in human liver tissue decreases with age [72, 78]. Defects of OXPHOS complexes III and IV were found in healthy tissue during cell aging and were more prevalent in cirrhotic livers [78].

### 4.4. Increased ROS Production

The free radical theory of aging was first hypothesized by Harman in 1956. He proposed that free radicals, due to the macromolecular damage they inflict, may be a potential cause of aging [49]. The main source of ROS is the RC in mitochondria, where superoxide radicals (O2-) are generated as byproducts, and the damage caused by them affects mtDNA [80]. Superoxide is known to be formed at seven potential sites in mitochondria, with complexes I and III showing the highest rates [81]. Since mtDNA is located in the mitochondrial matrix and protective structural proteins such as histones are absent, it is a more likely target for ROS than is nuclear DNA [51, 82]. In aged cells, the production of ROS is increased due to mitochondrial dysfunction, which then leads to activation of the recombination mechanism of mtDNA, thus increasing recombination errors, mtDNA deletions, and ROS production, resulting in a vicious cycle of mitochondrial damage [83, 84]. Prior studies in animal models have shown elevated ROS levels in heart mitochondria of old rats in contrast to young rats, which suggests that ROS play a pivotal role in the aging process [85]. Increased ROS production also promotes increased aortic stiffness, which is an early predictor of cardiovascular disease, highly associated with aging [86]. Young mice (4 months old) with high mitochondrial NADPH oxidase 4 (NOX4) expression, one of the major sources of ROS in the cardiovascular system, demonstrated a significant increase in aortic stiffness [86]. Furthermore, rats treated with rotenone, an inhibitor of complex I activity, showed increased levels of ROS production and damage to brain mitochondria [87]. Significantly increased ROS levels were also detected in mouse embryonic fibroblasts (MEFs) heterozygous for a null mutation of histone acetyltransferase 1 (Hat1^+/-^) [88]. The increased ROS might be the consequence of mitochondrial dysfunction, since Hat1, which is responsible for the acetylation of newly synthesized histone H4 on lysine 5 and 12 during chromatin assembly, affects mitochondrial function through the acetylation of mitochondrial protein and the acetylation status of mitochondrial proteins can be a key regulator of protein function [88]. It was shown that haploinsufficiency of Hat1leads to a significantly reduced lifespan of mice [88]. Premature aging was seen in Hat1^+/-^ mice, demonstrated by phenotypes such as early lordokyphosis (hunchback), muscle atrophy, minor growth retardation, reduced subcutaneous fat, cancer, and paralysis [88].

Kujoth et al. generated mutant mice with impaired proofreading activity of DNA-polymerase *γ* and measured H_2_O_2_ production in heart and liver mitochondria of young and old (3 months versus 9 months) mutant and wild-type mice. They observed no significant differences between young and old mice in terms of heart or liver mitochondrial function. Furthermore, assessment of protein carbonyls (a marker of oxidative damage to protein) and F2-isoprostanes (a marker of lipid peroxidation) showed no significant difference between mutant and wild-type mice [20]. Additionally, naked mole rats (the longest living rodent species) exhibit greater levels of accrued oxidative damage to lipids, DNA, and proteins than physiologically age-matched mice and equal to that of same-aged mice [89]. Taken together, these findings suggest that the role of free radicals in the process of aging remains unclear and further research on the subject is warranted.

### 4.5. Telomere Dysfunction

Telomeres are chromosome ends consisting of short-DNA 5′-TTAGGG-3′ repeats [90]. Their main function is to protect chromosome ends from being recognized as DNA damage, thus preventing their end-to-end fusion, recombination, and degradation [91]. Since DNA polymerases need templates to replicate DNA, telomerases add repeat sequences to the ends of chromosomes to avoid the shrinkage of linear chromosomes [90].

Telomere dysfunction affects mitochondrial biology [92]. The repression of the peroxisome proliferator-activated receptor-gamma coactivator (PGC) network, induced by telomere dysfunction, is associated with mitochondrial impairment as shown by compromised OXPHOS and respiration, decreased ATP generation capacity, and increased oxidative stress [92]. Guha et al. showed that mitochondrial dysfunction is a relevant factor in telomere shortening [93]. Cells with impaired mitochondrial function were found to be marked by higher levels of histone H4 at lysine 8 (H4K8) acetylation by hnRNPA2, a mitochondrial stress-responsive lysine acetyltransferase (KAT), and are associated with telomere attrition [93].

In summary, dysfunctional telomeres are linked to morphological and functional alterations in mitochondria, and telomere shrinking correlates with aging.

## 5. Role of Mitochondria in Intestinal Aging

Mitochondria and aging in postmitotic tissue, such as the heart, brain, and muscle, were discussed above. In contrast, the intestine is a mitotically active tissue and thus, its cells have the ability to proliferate and undergo a complete cell cycle [5]. Intestinal crypts in the intestinal epithelium contain replicating stem cells at their base, from which all cells within a crypt are derived [94]. Stem cell clones in the colon expand by dividing to form two daughter crypts; the process is known as crypt fission. mtDNA mutations affecting COX express the same phenotypic deficiency in COX in both arms of a crypt, and repeated crypt fission leads to patches of neighboring crypts deficient in COX. It was found that the size of these COX-negative patches increases with age due to the accumulation of mtDNA mutations [1, 5, 6, 23, 95].  Table 1 summarizes the results of the most relevant reviewed studies and their findings.

Taylor et al. examined human colonic crypt stem cells to determine the existence of mtDNA mutations in stem cells in normal tissue obtained during bowel resection or colonoscopy [23]. COX/succinate dehydrogenase (SDH) histochemical analysis revealed areas of COX-deficient or completely negative crypts throughout the colon specimens. Various subunits of complex IV were examined by immunohistochemistry. The mitochondrial-encoded subunits I and II showed the absence of immunoreactivity in COX-deficient crypts in all patient samples. The nuclear-encoded subunit IV showed a similar pattern, but to a lesser extent. To determine whether mtDNA mutations play a role in these COX-deficient crypts, real-time PCR and sequencing were performed to detect deletions and point mutations. No mtDNA deletions were seen, but several mtDNA point mutations were identified in COX-normal and COX-deficient crypts. The mutations in COX-normal crypts either were neutral polymorphisms or occurred at specific locations, probably causing an enzymatic defect but did not lead to protein degradation [23].

To investigate the effect of aging and the accumulation of mtDNA mutations in intestinal crypts on other OXPHOS complexes, Greaves et al. examined the expression levels of subunits of OXPHOS complexes I-IV by histochemistry and immunohistochemistry. They used colonic mucosal samples from 20 patients aged 18-84 years and evaluated colonic crypts. 11.2% of the crypts showed no or reduced expression of one or more complexes, and the frequency increased exponentially with age. Isolated reductions in the expression levels of complex I and complex IV subunits were noted, but no isolated complex III deficiency was found. Also, the genetic basis of reduced expression levels in different subunits of the RC complex was evaluated by sequencing the entire mitochondrial genome of cells that had defective expression of either single or multiple complexes (2 cells with complex I deficiency only and 16 cells with combined complex I, III, and IV deficiencies). A number of point mutations affecting mitochondrial-encoded RC subunits were observed [5, 23].

OXPHOS complexes are known to form supercomplexes and do not only exist as single complexes. Thus, complexes and supercomplexes coexist in the mitochondrial inner membrane and multiple pathways coordinate their assembly, depending on different tissue and cellular metabolic status [96–99]. The existence of supercomplexes could be one reason for multiple complexes being affected by mutations in protein-coding genes. A mutation in the cytochrome b gene, a subunit of complex III, affects the expression levels of not only complex III but also complex I. However, a mutation in a subunit of complex I does not influence the stability of complex III [100]. Thus, isolated reduction of complex I but not complex III has been observed in human colonic crypt mitochondria [5, 101].

To study OXPHOS complexes in the intestinal mucosa during the aging process, Özsoy et al. recently investigated subunits of OXPHOS complexes I-V and the voltage-dependent-anion-selective channel 1 protein (VDAC1, porin) in biopsies of intestinal mucosa with no endoscopic or histomorphologic abnormalities (i.e., no inflammation or alterations in crypt architecture) by immunohistochemistry [102]. Formaldehyde-fixed paraffin-embedded tissue samples from both the small and large intestines of 55 patients aged 4-82 years, thus including pediatric individuals, were analyzed. In addition, comparisons between material from different intestinal segments (terminal ileum, ascending colon, and sigmoid colon/rectum) were made. In contrast to previous studies, Özsoy et al. applied a scoring system to characterize the overall expression level of each sample, which involved multiplication of the staining intensity by the percentage of positively stained cells. Furthermore, the numbers of positive-, partial positive-, and negative-stained intestinal crypts were determined. The authors found that the protein expression levels of OXPHOS complexes increased from childhood into adulthood and then decreased in elderly individuals, while the numbers of crypts with partial or complete loss of expression of complexes I and IV increased continuously with age. These data suggest that the continuous decline in the levels of mitochondrial OXPHOS complexes in crypts might be compensated in adulthood but ultimately leads to reduced expression levels in elderly persons, which become evident beyond the age of 60 years. In clinical terms, these findings raise two questions: (1) can the process of aging be delayed by targeting mitochondrial pathways with therapeutics? and (2) pathophysiologically, are these findings associated with disorders of the intestinal mucosa, e.g., inflammation [102]?

mtDNA mutations also affect the morphology of the small intestine [31]. mtDNA-polymerase *γ* (PolgD257A) mutated mice were examined for structural changes in the crypts of Lieberkühn. In PolgD257A mice, the small intestine was significantly enlarged and there was a 10-fold increase in apoptosis within the crypts, containing both intestinal stem cells and progenitor cells, compared to wild-type mice. Additionally, cell migration along the crypt-villus axis was significantly decreased in mutated mice and they also demonstrated a significant decline in lipid absorption, leading to increased fecal excretion of lipids, compared to wild-type mice [31].

Regarding mitochondrial structure in the aging process, the midgut epithelium of aged *Drosophila melanogaster* was found to exhibit mitochondria with disarranged cristae and also crista-free areas [24]. Additionally, the mitochondria showed accumulation of dense bodies, virus-like particles, and fiber-shaped and reticular structures [24].

Regarding the role of oxidative stress in aging of the intestine, a significant age-related increase of 8-hydroxyguanosine levels in human colorectal biopsy samples was shown [103]. However, as mentioned above, the impact of free radicals on the aging process is ambiguous, and more research in this field is required.

As discussed earlier, shortening of telomere length is thought to be a crucial factor in aging. Late-generation (G3) telomerase-deficient (mTERC^−/−^) mice were analyzed for basal and stimulated duodenal bicarbonate (HCO_3_^−^) secretory rates, and these rates were found to be reduced in comparison to those of age-matched wild-type mice [104]. Also, morphologic changes to the duodenal mucosa, including slimming and shortening of villi, were observed [104]. P21, encoded by Cdkn1a, is a downstream effector of telomere shortening-caused aging [105]. Deletion of this cyclin-dependent kinase inhibitor (in mTERC^−/−^ p21^−/−^ double knockout mice) rescued the villus atrophy of the duodenal mucosa and normalized HCO_3_^−^ secretion rates [104]. These findings suggest that telomere shortening induces an imbalance between harmful and protective secretory products in the duodenum, leading to an increased risk of ulcer formation [104].

Aging was also found to be associated with increased serum levels of acute-phase proteins and proinflammatory cytokines, including interleukin-6 (IL-6) and tumor necrosis factor alpha (TNF-*α*), contributing to the theory of inflammaging [106]. Tran and Greenwood-Van Meerveld compared colon biopsies from old baboons to young ones; old baboons had greater expression of interferon gamma (IFN-*γ*), IL-6, and IL-1*β*, linked to a disruption of intestinal permeability [25]. However, in humans, there was upregulation of IL-6 in terminal ileum biopsies, but no effects on IFN-*γ*, TNF-*α*, and IL-1*β* expression were noted in the elderly compared to young persons, and ileal permeability to macromolecules was unchanged [26]. Additionally, IL-6 was shown to have an impact on mitochondrial function [107, 108]. In a rat model, Lowes et al. demonstrated that antioxidants which protect mitochondria (MitoQ, MitoE, or melatonin) also lower IL-6 levels and oxidative stress, thereby improving mitochondrial activity and reducing organ dysfunction in the case of acute sepsis [107]. In chronic alcohol consumption by mice, mtDNA damage was seen and IL-6 was shown to be important for the recovery of the liver from mtDNA oxidation. IL-6 knockout and wild-type mice were fed ethanol for 4 weeks, which led to mtDNA injury in both groups. In contrast to IL-6 knockout mice, wild-type mice were able to activate repair mechanisms and avoid mtDNA mutations [108].

### 5.1. Clinical Aspects

Recent data suggest that mitochondrial dysfunction compromises intestinal barrier function, leading to increased gut permeability and favoring invasion by immune cells [26]. Elevated IL-6 levels were observed in aged intestine, which was followed by an increase of claudin-2 expression, resulting in a decline in transepithelial electrical resistance [26]. Mitochondrial dysfunction is also an early sign of inflammation [109]. Xue et al. observed that wild-type mice treated with dextran sodium sulfate (DSS, a compound that induces intestinal inflammation) showed mitochondrial changes, including swelling, decreased ATP production, increased ROS generation, and increased antioxidant protein expression in colonic epithelial cells after less than 3 days of treatment; tissue damage and cytokine induction occurred at day 7 [109].

Frailty is a syndrome affecting elderly persons, characterized by increased vulnerability to minor stressors and resulting in a higher risk of disability, hospitalization, falls, and mortality [110]. In addition, constipation is also known to be more frequent in persons with frailty than in robust controls [32–34]. Mitochondrial impairment might play a role in this age-associated syndrome, possibly linked to mitochondrial genetic variation [12]. Cu/Zn superoxide dismutase knockout (Sod1KO) mice showing weight loss, weakness, low physical activity, and exhaustion were used as a model of frailty. A high degree of oxidative damage to DNA was noted in the liver of these mice [111]. Additionally, the skeletal muscle of the mice showed mitochondrial functional alterations, including increased ROS and decreased energy production [111]. Oxidative damage is a pivotal factor in the etiology of frailty syndrome. Despite many studies suggesting a correlation between ROS and aging, recent data show that oxidative damage is associated with frailty rather than chronological aging [112, 113]. The plasma of frail individuals was found to contain significantly higher levels of oxidative damage markers (e.g., malondialdehyde and 4-hydroxy-2,3-nonenal- (HNE-) protein adducts) compared to nonfrail patients [114]. Furthermore, Wu et al. showed a correlation between increased oxidative stress markers, serum 8-hydroxy-2′-deoxyguanosine, and frailty status in elderly persons [115].

The association of mitochondrial alterations and intestinal dysmotility was described in patients with mutations in the nuclear-encoded polymerase *γ* (POLG1) and mitochondrial tRNA genes (e.g., the mitochondrial tRNA^Val^ (MTTV) gene), who presented with MNGIE-like clinical signs and symptoms, including cachexia and intestinal dysmotility [36]. MNGIE is an autosomal recessive disease caused by mutations in the thymidine phosphorylase (TYMP) gene [116]. Clinical manifestations include ptosis, progressive external ophthalmoplegia, gastrointestinal dysmotility, cachexia, peripheral neuropathy, and leukoencephalopathy [117]. The condition where leukoencephalopathy is absent on brain MRI and no abnormalities of the TYMP gene are present is termed MNGIE-like disorder [118].

Existing data support the notion that increasing age is also associated with deterioration of intestinal motility [32–34]. Old monkeys exposed to barium-impregnated polyethylene spheres via orogastric intubation demonstrated a significant decline in intestinal motility compared to young monkeys [32]. Radiographs taken at different time intervals showed a delay in marker elimination in aged animals [32]. Also, constipation, which is frequently seen in the elderly population, is a result of intestinal dysmotility, increased intake of constipating drugs, and less exercise [33, 34].

Additionally, it is thought that oxidative stress plays a role in colonic dysmotility [35]. Mice exposed to oxaliplatin, a chemotherapeutic agent known for its gastrointestinal side effects such as nausea, vomiting, bloating, diarrhea, and/or constipation, showed significant enteric neuronal loss, increased ROS production and mitochondrial membrane depolarization, and increased permeability [35]. Furthermore, the mice exhibited alterations in neuromuscular transmission and intestinal smooth muscle tone, resulting in colonic dysmotility and chronic constipation [35].

## 6. Conclusion

Aging is associated with a decline in energy production at the cellular level caused by mitochondrial alterations. This has been demonstrated in several animal models as well as in human tissue [5, 7, 8, 20–23, 67, 69, 72]. Owing to its high energy consumption, the human colon is markedly affected by the process of aging. mtDNA mutations accumulate in human colonic crypts over time, causing deficiencies and defects in the RC complexes [5]. Thus, mitochondrial disturbances and increased ROS production are believed to be a potential cause of intestinal aging. Manifestations of intestinal aging include disturbances of the intestinal barrier and motility [25, 26, 32, 33, 35]. In addition to the mitochondrial alterations that accompany the pathophysiological sequelae of aging, other disorders of the human colon are associated with mitochondrial dysfunction. However, further investigation is necessary to unravel the link between mitochondrial alterations and intestinal aging, the connection to intestinal barrier disturbances, and other bowel diseases, as well as the potential therapeutic implications, e.g., treatment with antioxidant agents.

## Figures and Tables

**Table 1:** Most relevant studies regarding the role of mitochondria and their alterations in the aging intestine.

Reviewed study	Model	Organ	Cell	Observed findings/alterations
Anton-Erxleben et al. 1983	Drosophila melanogaster	Midgut	Epithelial cells	The midgut epithelium of aged *Drosophila melanogaster* was found to exhibit mitochondria with disarranged cristae and also crista-free areas. Additionally, mitochondria showed accumulation of dense bodies, virus-like particles, and fiber-shaped and reticular structures.
Taylor et al. 2003	Human	Colon	Crypt stem cells	COX deficiency or complete crypt loss throughout the colon specimens. Mitochondrial-encoded subunits I and II of complex IV showed absence of immunoreactivity in COX-deficient crypts in all patient samples. The nuclear-encoded subunit IV showed a similar pattern, but to a lesser extent.
Greaves et al. 2010	Human	Colon	Mucosal cells	11.2% of the crypts showed no or reduced expression of one or more complexes, and the frequency increased exponentially with age. A number of point mutations affecting mitochondrial-encoded RC subunits were observed.
Zhang 2010	Mice	na	na	In chronic alcohol consumption in mice, mtDNA damage was seen and IL-6 was shown to be important for the recovery of the liver from mtDNA oxidation. IL-6 knockout and wild-type mice were fed with ethanol for 4 weeks, which led to mtDNA injury in both groups. In contrast to IL-6 knockout mice, wild-type mice were able to activate repair mechanisms and avoid mtDNA mutations.
Fox et al. 2012	Mice	Small intestine	Intestinal stem cells, progenitor cells	mtDNA-polymerase *γ* (PolgD257A) mutated mice were examined for structural changes in the crypts of Lieberkühn. Significant enlargement and 10-fold increase in apoptosis in the small intestine. Significantly decreased migration along the crypt-villus axis and significant decline in lipid absorption, leading to increased fecal excretion of lipids, compared to wild-type mice.
Tuo et al. 2012	Mice	Duodenum	Mucosal cells	Reduced basal and stimulated duodenal bicarbonate secretory rates in late-generation (G3) telomerase-deficient (mTERC^−/−^) mice in comparison to those of age-matched wild-type mice. Slimming and shortening of villi of the duodenal mucosa. Telomere shortening induces an imbalance between harmful and protective secretory products in the duodenum, leading to an increased risk of ulcer formation.
Lowes et al. 2013	Rats	na	na	Antioxidants which protect mitochondria (MitoQ, MitoE, or melatonin) also lower IL-6 levels and oxidative stress and thereby improving mitochondrial activity and reducing organ dysfunction in the case of acute sepsis.
Tran and Greenwood-Van Merced 2013	Baboons	Colon	Epithelial cells	Old baboons had greater expression of interferon gamma (IFN-*γ*), IL-6, and IL-1*β*, linked to a disruption of intestinal permeability.
Man et al. 2015	Human	Terminal ileum	Epithelial cells	Upregulation of IL-6 in terminal ileum biopsies, but no effects on IFN-*γ*, TNF-*α*, and IL-1*β* expression were noted in the elderly compared to young people, and ileal permeability to macromolecules was unchanged.
Özsoy et al. 2020	Human	Colon	Mucosal cells	The protein expression levels of OXPHOS complexes increased from childhood into adulthood and then decreased in elderly individuals, while the numbers of crypts with partial or complete loss of expression of complexes I and IV increased continuously with age.
